# Unsupervised Machine Learning Driven Analysis of Verbatims of Treatment-Resistant Schizophrenia Patients Having Followed Avatar Therapy

**DOI:** 10.3390/jpm13050801

**Published:** 2023-05-06

**Authors:** Alexandre Hudon, Mélissa Beaudoin, Kingsada Phraxayavong, Stéphane Potvin, Alexandre Dumais

**Affiliations:** 1 Centre de Recherche de l’Institut Universitaire en Santé Mentale de Montréal, Montreal, QC H1N 3J4, Canada; 2 Department of Psychiatry and Addictology, Faculty of Medicine, Université de Montréal, Montreal, QC H3T 1J4, Canada; 3 Faculty of Medicine and Health Sciences, McGill University, Montreal, QC H3G 2M1, Canada; 4 Services et Recherches Psychiatriques AD, Montreal, QC H1C 1H1, Canada; 5 Institut National de Psychiatrie Légale Philippe-Pinel, Montreal, QC H1C 1H1, Canada

**Keywords:** psychotherapy, virtual-reality therapy, auditory hallucinations, schizophrenia, machine learning

## Abstract

(1) Background: The therapeutic mechanisms underlying psychotherapeutic interventions for individuals with treatment-resistant schizophrenia are mostly unknown. One of these treatment techniques is avatar therapy (AT), in which the patient engages in immersive sessions while interacting with an avatar representing their primary persistent auditory verbal hallucination. The aim of this study was to conduct an unsupervised machine-learning analysis of verbatims of treatment-resistant schizophrenia patients that have followed AT. The second aim of the study was to compare the data clusters obtained from the unsupervised machine-learning analysis with previously conducted qualitative analysis. (2) Methods: A k-means algorithm was performed over the immersive-session verbatims of 18 patients suffering from treatment-resistant schizophrenia who followed AT to cluster interactions of the avatar and the patient. Data were pre-processed using vectorization and data reduction. (3): Results: Three clusters of interactions were identified for the avatar’s interactions whereas four clusters were identified for the patient’s interactions. (4) Conclusion: This study was the first attempt to conduct unsupervised machine learning on AT and provided a quantitative insight into the inner interactions that take place during immersive sessions. The use of unsupervised machine learning could yield a better understanding of the type of interactions that take place in AT and their clinical implications.

## 1. Introduction

Schizophrenia is a severe mental illness that affects millions of people worldwide and can profoundly impact the affected individual, their families, and society as a whole [1,2,3]. Chronic psychotic symptoms can make it difficult for individuals with the illness to maintain relationships, hold a job, and have a fulfilling life [3]. Moreover, living with schizophrenia leads to a significantly reduced life expectancy due to a much higher risk of completing suicide and suffering from chronic physical conditions such as cardiovascular diseases or diabetes [4,5]. The societal burden of this illness is quite high given the loss of productivity and the substantial costs associated with treating schizophrenia (i.e., hospitalizations, regular healthcare appointments, and medications) [2,6]. Most distressing acute symptoms can be substantially reduced using antipsychotic medications; however, up to a third of patients fail to improve, making them resistant to treatment [7]. These individuals often have a poorer quality of life, experience more frequent hospitalizations, have higher rates of suicide, leading to significantly higher societal costs compared to those who respond appropriately to antipsychotics [8]. The most effective medication for this condition, clozapine, is not always an option since it has poor tolerability and requires careful monitoring for severe side effects [9,10]. Moreover, a significant subset of treatment-resistant patients also fails to respond to clozapine; these are often referred to as being “ultra-resistant” to treatment [11].

To learn how to cope with their persistent symptoms, patients with treatment-resistant schizophrenia are generally encouraged to undergo psychotherapy [12]. The most prevalent and distressing symptom is auditory verbal hallucinations (AVH) i.e., hearing voices); therefore, this specific component of schizophrenia is targeted by a few psychotherapeutic approaches [13,14]. The most studied and widespread one is cognitive–behavioral therapy, which has been shown to be significantly more effective than a control condition in reducing the frequency and distress associated with AVH in this population [15]. However, the effect size is only moderate and the symptoms of only a small subset of patients are reduced in a clinically significant manner [16,17,18,19]. Additionally, according to a recent meta-analysis, CBT for psychosis might have little to no impact on quality of life [19]. This could be due to the fact that this therapy, largely based on psychoeducation and mindfulness, does not offer the patient an opportunity to practice interacting with their voices and finding new coping strategies under therapeutic supervision. To address this gap, a few novel therapeutic approaches are now focused on having the patient improve their relationship with their voice(s), notably by entering a dialogue with them [20]. This can be achieved using different techniques such as chairwork (i.e., having the patient take the role of the voice in one chair and then answering them in a different chair), through role-play with the therapist, or by dialoguing with an avatar representing the distressing voice [20]. Avatar therapy (AT), which was initially developed using an avatar on a 2D screen, has now been adapted to virtual reality (VR), thereby increasing the immersive aspect of psychotherapy [18,21,22]. In this therapy, patients with treatment-resistant schizophrenia are first invited to create and personalize an avatar resembling the mental image that they have of their most distressing hallucination, both in terms of physical appearance and tone of voice. Afterward, patients undergo six to ten one-hour weekly therapeutic sessions which all include approximately 5 to 20 min of dialogue with their avatar in VR. The avatar is animated by the therapist, who is installed in a separate room and has their voice modified in real-time. In addition to role-playing the voice, the therapist also has control over the facial expressions as well as the distance between the avatar and the patient. During the first few sessions, the therapist starts by repeating verbatim what the patient reports that their voice usually says, and mostly uses provocative techniques. For example, the therapist, animating the avatar, might repeat “you are worthless”. However, the avatar gradually opens to the patient and starts using more and more positive techniques [18,21,22]. The different themes addressed during AT have been described in detail in a previous qualitative study by Beaudoin and her team [23]. Notably, the avatar mainly used techniques that were classified as provocative (e.g., threats, accusations, affirmations of omnipotence) or positive (e.g., reinforcement, empathetic listening). The patients responded in a few different ways: with an emotional response (positive, neutral, or negative), by mentioning beliefs about the voices and/or schizophrenia (e.g., omnipotence, malevolence), self-perceptions (i.e., self-appraisal or self-deprecation), coping mechanisms (e.g., self-affirmation, counterattack), or aspirations (e.g., prevention strategies) [23,24].

While previous qualitative studies highlight promising avenues to better comprehend the inner psychotherapeutic processes that might be linked to a positive outcome, it is possible that some elements are underexamined or prone to subjective biases, which are prevalent in such studies [25]. The use of artificial-intelligence-driven approaches, such as unsupervised machine learning, is an increasingly seen technique in various medical fields in order to derive objective data from several types of textual datasets (and other sources of datasets) in the medical field [26]. It is a technique in which unlabeled data are used to conduct different types of tasks such as hierarchical learning, data clustering, latent variable modeling, dimensionality reduction (on large datasets), and outlier detection [27]. A few implementations of such algorithms are found in psychiatry. For example, recent research conducted by Kung et al. (2022) used unsupervised learning to identify qualitative subtypes of depression based on the clinical data from 18,314 patients with depression [28]. Another recent example is the identification of five subgroups of psychosis amongst 765 individuals suffering from *DSM-IV* diagnoses of schizophrenia, bipolar affective disorder (I/II), schizoaffective disorder, schizophreniform disorder, and brief psychotic disorder by using clustering methods: affective, suicidal, high functioning, depressive, and severe psychosis [29]. In the field of psychotherapy and psychotherapeutic approaches, the latest literature review on the subjective identifies nine studies that used unsupervised machine learning [30]. Most of these applications were used to perform human-like responses to interact with patients after learning from datasets of multiple interactions derived from thousands of therapy sessions. An example of such application is the development of *ClientBot*, by Tanana et al. (2019), which used natural language-processing methods for automated coding rather than human coders to perform interactions with the patients [31]. To our knowledge, the use of unsupervised machine learning to objectively assess verbatims from AT has never been conducted. Natural-language-processing (a subset of machine learning) approaches for patients suffering from schizophrenia are currently being studied and demonstrate promising avenues to characterize sub-clinical linguistic differences in schizophrenia-spectrum disorders which might be clinically relevant [32]. Analysis of verbatims using unsupervised learning might therefore provide insights as to different types of interactions taking place during the immersive sessions.

This study’s primary aim was to conduct an unsupervised machine-learning analysis of verbatims of treatment-resistant schizophrenia patients that had followed AT. The second aim of the study was to compare the data clusters obtained by the unsupervised machine-learning analysis with the main themes identified by Beaudoin et al. (2021) through human-driven qualitative analysis. The hypothesis was that unsupervised machine-learning analysis will provide clusters similar to the main themes identified by Beaudoin et al., while providing insight as to how certain themes might be sub-divided.

## 2. Materials and Methods

### 2.1. Participants and Recruitment

The participants included in this study received AT as part of pilot trials at the Centre de recherche de l’Institut universitaire en santé mentale de Montréal (CR-IUSMM) and one ongoing trial comparing AT to CBT. The participants all belonged to the clinical trial registered on Clinicaltrials.gov (identifier number: NCT03585127) [18,21]. Included participants received nine psychotherapeutic sessions, of which eight were immersive. In these sessions, the patients interacted with an avatar representing their most significant AVH. The participants included in this study were all patients at the IUSMM, over 18 years of age, who were suffering from treatment-resistant schizophrenia as defined by the absence of response to two or more antipsychotics, and who had received AT between 2017 and 2020. The ethics committee of CR-IUSMM approved the study as part of the protocol for AT.

### 2.2. Data Collection

First, a content analysis was performed on 125 immersive sessions (1419 min of therapy) from 18 patients with treatment-resistant schizophrenia who underwent avatar therapy in the context of either one of two clinical trials assessing the efficacy of this therapy [24]. Each therapy session was first transcribed (Canadian French), and then read and carefully annotated by each member of the research team. Discussions then took place every week to identify each theme and organize them hierarchically into a grid. Then, each verbatim (i.e., a group of sentences representing one expressed idea) was coded into one of the identified themes. Transcripts were annotated sequentially, and the grid was adjusted as the coding progressed in a back-and-forth manner, and the process only stopped when data saturation occurred (i.e., when the therapies of a few participants were coded without having to adjust the grid). To assess potential inter-rater variability, 63% of the sample was also coded by a second person; overall, the inter-rater agreement was fair for the detailed theme grid (Scott’s Pi = 0.514) and moderate for agreement on the key themes only (Scott’s Pi = 0.660). More details about the methodology and the results of this analysis can be found in a previous paper published on that matter [24].

**Figure 1:**
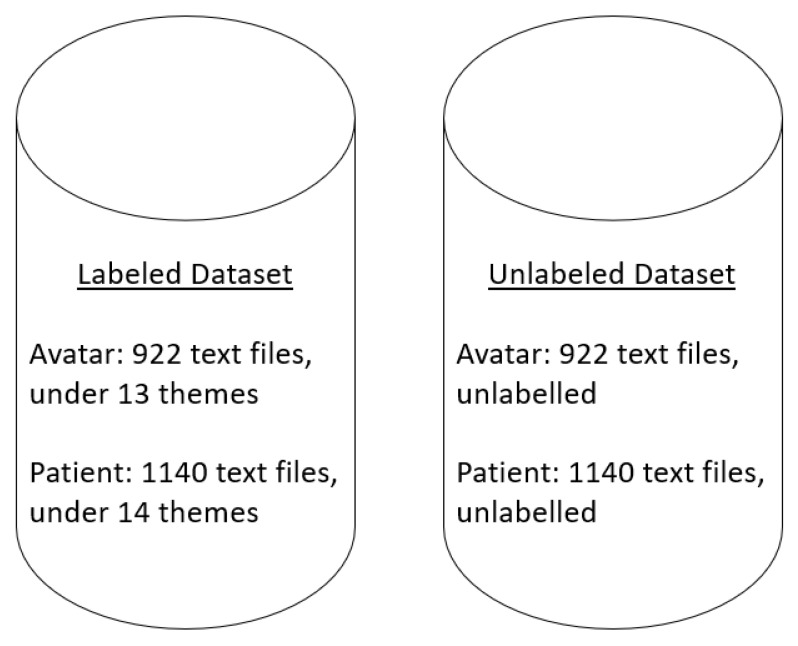
Datasets. The datasets contained text files of interactions between the avatar and the patient from the verbatims of immersive sessions. Each text file contained from 1 to 40 interactions. In the labeled dataset the text files are categorized as per one of the sub-themes of Beaudoin et al. (2021), and in the unlabeled dataset, the text files are not categorized.

From the above content analysis, two datasets were developed. The first dataset contained all the labeled interactions for the avatar and the patient, whereas the second dataset contained unlabeled interactions for the avatar and the patient as per Figure 1. In the labeled dataset, Beaudoin et al. (2021) identified two major categories of interactions for the avatar: *confrontational techniques* and *positive techniques*. For the patient, they identified five categories: *self-perceptions*, *aspirations*, *emotional responses*, *coping mechanisms*, and *beliefs about voices and schizophrenia*.

### 2.3. Data Analysis

**Figure 2:**
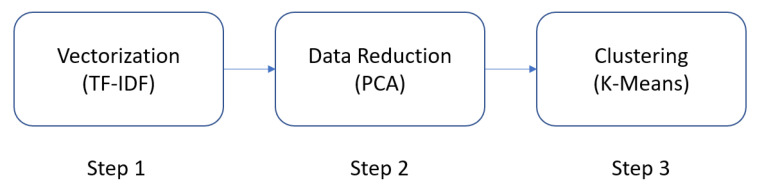
Overview of the steps performed to cluster the unlabeled data.

The various steps included in the data analysis are presented below. The overall flow of the data analysis process is presented in Figure 2.

#### 2.3.1. Unsupervised Machine-Learning Algorithm

##### Implementation

A k-means clustering algorithm was used to conduct the clustering of the data from the dataset containing the unlabeled interactions using Python 3.9 with the Scitkit-Learn open library [33,34]. This widely used algorithm attempts to cluster similar data in an easy-to-interpret, relatively fast, and scalable way while guaranteeing convergence of the data [35]. It determines whether two items are identical and clusters them based on their Euclidean distance, representing the length of a line traced between two data points [36]. The number of clusters is determined in advance, and the following steps are performed iteratively [37]. First, the center of each cluster (centroid) is randomly selected, then the Euclidean distance of all data points to the centroids is calculated, and the data points are then assigned to the closest cluster. Then, the new centroid of each cluster is identified by taking the mean of all data points in the cluster and repeating the process until all the points converge and the cluster centers stop moving.

To determine the number of clusters, an elbow plot is used. This technique illustrates the global dissimilarity (also known as inertia) between the data points and the number of potential clusters. Dissimilarity refers to the squared Euclidean distance between the data points and the cluster centers, and global dissimilarity is, therefore, the sum of dissimilarity for all the data points within all the clusters [38]. The use of an elbow plot as compared to other techniques (i.e., Silhouette’s coefficient and the gap statistic) was because of the smaller size of the dataset, the notion that the data might not be clearly separated, and the gain in time complexity [39].

### 2.4. Data Preprocessing

The term frequency-inverse document frequency statistic (TF-IDF) was used to convert the raw text of all the textual interactions into numerical vectors to be used by the k-means algorithm. Therefore, all the sentences of each text file included as part of the dataset are converted into a vector. This step is necessary because length between raw textual data points cannot be measured and compared [40]. Considering the wide variety of interactions that are taking place in AT and the previous knowledge of the qualitative insights of these interactions, these textual interactions were assumed to be linearly separable. The TfidfVectorized of the Scitkit-Learn open library was used [33].

### 2.5. Data Reduction

A principal component analysis (PCA) using the Scitkit-Learn open library was conducted on the vectorized data prior to the k-means analysis [33]. Reducing the dimensionality of a dataset is a method performed to increase interpretability while minimizing information loss [41]. PCA is among the most used algorithms for such tasks as it attempts to estimate the linear combinations of the different independent variables by creating uncorrelated variables (principal components) that will successively maximize variance. It accomplishes this by locating a collection of orthogonal vectors known as the principal components, which reflect the directions in which the data’s largest variation occurs. Each consecutive principal component is chosen to be orthogonal to the previous ones and to capture the next biggest amount of variance. The first principal component corresponds to the direction with the largest amount of variation. Fewer dimensions and frequently easier analysis and visualization characterize the resulting converted dataset.

### 2.6. Comparing the Unsupervised Machine-Learning Clustering with the Labeled Data

A descriptive statistical analysis of the comparison between the previously labeled data and the clustered labeled data was performed. This was done using a simple Python 3.9 program that remapped all the unlabeled interactions from the unlabeled dataset with their labeled counterpart while keeping track of their newly identified cluster. As per Beaudoin et al. (2021), the frequency of each sub-theme was compared between both datasets.

## 3. Results

Vectorization and data reduction were successfully conducted for all the data points of the unlabeled dataset prior to performing clustering. Interactions from 922 text files were identified for the avatar and 1140 text files for the patient.

### 3.1. Clustering

**Figure 3:**
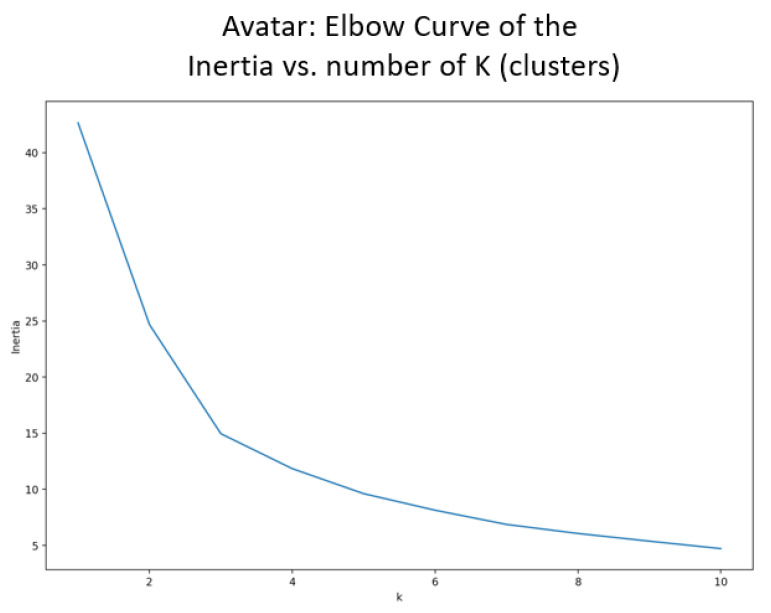
Elbow curve to identify the number of clusters for the avatar interactions.

It can be observed in Figure 3 that the avatar elbow curve indicates that the optimal number of clusters should be between two and four. Therefore, three clusters were selected as the initiation parameter for the k-means algorithm.

**Figure 4:**
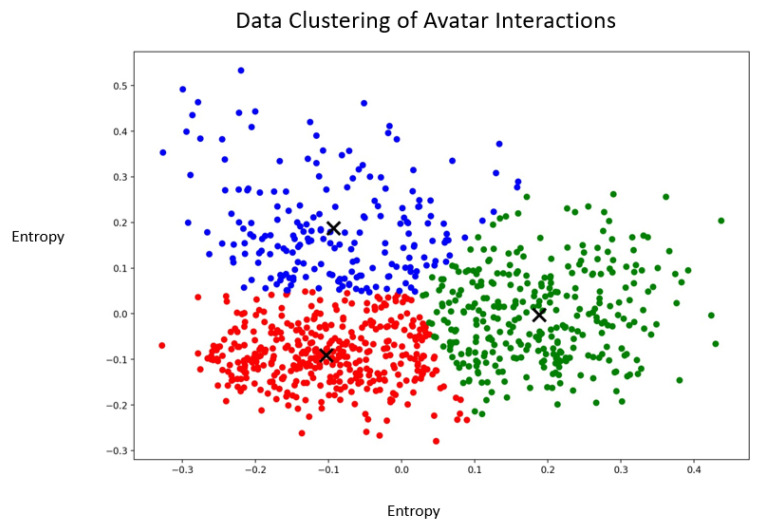
Graphical representation of the clusters containing the vectorized interactions of the avatar. The X represent the centroids of the clusters. The blue, green and red colors represent the different clusters.

As displayed in Figure 4, data points were scattered across the three different clusters. The red cluster appeared to have more homogeneous data points, whereas the blue cluster had data that were very far apart and more heterogeneous. In the middle of the graph, there appeared to be no clear delimitation across the three clusters which might have indicated that these data points were not clearly divisible into different clusters. These interactions could likely be susceptible to various diverging interpretations if they were to be qualitatively assessed by human coders.

Examples of verbatims from the different clusters can be found below (translated from Canadian French to English):

*Blue cluster:*

“You are supposed to let me win.”

“They are right, you are the one that stole it.”

“I don’t believe you; you can’t be right.”

*Green cluster:*

“Do you believe in yourself?”

“Maybe you are becoming crazy? Are you?”

“Let’s make peace.”

*Red cluster:*

“How will you do it? What is it that you will do?”

“Do you want me to stay? Should I leave?”

“What could they do for you?”

**Figure 5:**
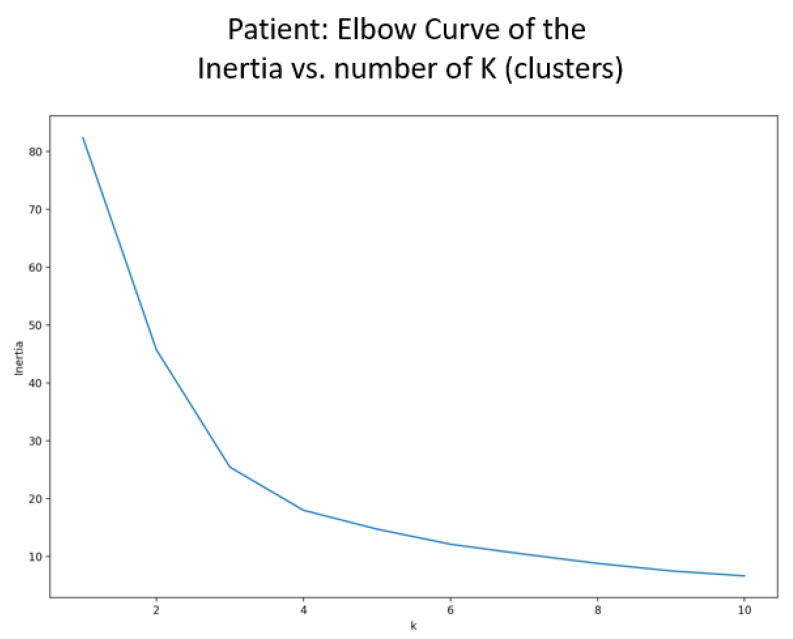
Elbow curve to identify the number of clusters for the patient interactions.

As depicted in Figure 5, the patient elbow curve indicated that the optimal number of clusters should be around four. Therefore, four clusters were selected as the initiation parameter for the k-means algorithm.

**Figure 6:**
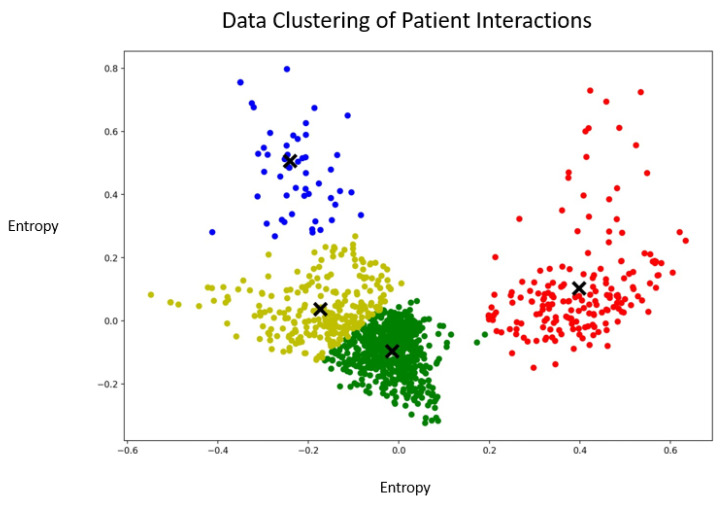
Graphical representation of the clusters containing the vectorized interactions of the patients. The X symbols represent the centroids of the clusters. The blue, green and red colors represent the different clusters.

As displayed in Figure 6, data points were scattered across the four different clusters. The yellow and green clusters appeared to overlap, whereas the red and blue clusters were well delimited from all the other clusters. This indicated that some interactions clearly belonged together, whereas it was difficult to discriminate between interactions belonging to the yellow and the green clusters. The green cluster had very homogenous interactions, whereas the blue and red clusters were heterogeneous.

Examples of verbatims from the different clusters can be found below.

*Blue cluster:*

“I have weaknesses.”

“You are right, I need to call my mother. It is important that I call her very soon.”

“Yes, it is a fact. I’m not a good person.”

*Yellow cluster:*

“I’d like you to stop talking to me.”

“No, you can’t. You are not allowed to do this to me.”

“I would like you to give me positive energy and please stop trying to destroy me all the time.”

*Green cluster:*

“Life is great, my friend.”

“You are not so much in my head anymore.”

“This week you left me alone. I like that.”

*Red cluster:*

“I’ll confront you and tell you that you are very ill.”

“I need to stop playing slot machines.”

“The doctor is helping me. He is my ally. He is telling me what to do.”

### 3.2. Comparison with Previously Labeled Data

Cross-labeling of the unlabeled dataset with the labeled dataset was conducted. Table 1 presents the original division of the text files and their classification (labels) for the labeled dataset, whereas Table 2 displays textual entities’ mapping and their classification per the unlabeled dataset. Compared to Beaudoin et al. (2021), the clustering analysis identified three clusters (labels) for the avatar interactions and four clusters for the patient interactions.

With the mapping of the labels on the clustering data, it can be observed for the avatar that some of the confrontational techniques appear to have been shared across the blue and green clusters. In contrast, the positive techniques were mostly spread across the green and the red clusters. Clustering highlights the heterogeneity of the interaction across these categories previously defined as *confrontational techniques* and *positive techniques*.

As for the patient, most of the interactions previously defined as per the five labels appear to have been clustered into the green and yellow clusters, especially *emotional responses* in the yellow cluster. They mostly regroup interactions that were previously classified as *coping mechanisms*, *aspirations, and beliefs about voices and schizophrenia*. The blue and red clusters appear to regroup interactions that were mainly scattered across the previously defined labels. Interactions previously labeled as *coping mechanisms* appear to be less present in the blue cluster, whereas they were more prevalent in the red cluster. The opposite classification can be observed with the interactions previously labeled as *aspirations*.

## 4. Discussion

The main goal of this study was to conduct unsupervised machine-learning analysis verbatims of treatment-resistant schizophrenia patients that had followed AT. This was done by vectorizing textual interactions of the avatar and the patient during immersive sessions of AT, reducing the complexity of the data, and performing a cluster classification of unlabeled data. That enabled the identification of three clusters for the avatar’s interactions and four clusters for the patient’s interactions. These unlabeled clustered data were then remapped as per the previous qualitative study on the same verbatims Beaudoin et al.

It was possible to observe three distinct clusters for the avatar interactions. Considering the variety of potential interactions that the therapist must employ during the immersive session to personalize the experience for each patient, it is possible that this provides a distinction between the *confrontational techniques* classified in the blue cluster and those in the red cluster. As indicated in O’Brien et al. (2021), in AT, the therapist must consider a formulation to inform the direction of the therapy as well as quickly responding as the characterized avatar [42]. Several studies outline the use of direct confrontation in psychotherapy as well as empathetic confrontation. Empathic confrontation is often observed as part of schema therapy to address patients’ maladaptive behaviors and it also serves emotional regulation [43]. On the other hand, direct confrontation can be seen in AT to provoke the patient by mimicking their experience with AVH. Since these two techniques differ in terms of interactions and delivery, this might explain the division of these interactions between two clusters. As for *positive techniques*, they were also scattered across two clusters (red and green). A previous study on integrative psychotherapy indicated that therapeutic alliance had the most evidence as a predictor of patient change [44]. One challenge of AT is, therefore, to bring forward the personification of the AVH while maintaining the therapeutic alliance and inducing positive changes, which may imply different types of positive techniques. In CBT, psychotherapeutic approaches for patients with schizophrenia include the development of trust, normalization, coping strategy enhancement, and reality testing. In the green cluster, some of the interactions previously classified as *positive techniques* appear to partly include elements of *confrontational techniques*. That might be part of reality testing, which might appear confrontational while potentially assessing the self-perceptions or beliefs of the patient about their AVH.

The patient interactions were classified into four different clusters, meaning that the interactions might have been less heterogenous than what was found in previous qualitative study on the same dataset [23]. The blue cluster contained very few interactions, which suggests there were outliers in the interactions between the patients and their avatars. A recent study assessing 499 language samples with a natural language processing algorithm on patients with schizophrenia or bipolar disorder outlined that sociodemographic and individual differences should be considered while conducting language analysis for psychosis [45]. These, as well as relationships with others, were not specifically captured with the previously conducted qualitative analysis. That might account for the outliers identified in the blue cluster, but further analyses should be conducted. Most of the *coping mechanism* interactions were found in the green cluster, whereas the *emotional responses* were found in the yellow cluster while these two clusters seemed to intersect. That is not surprising considering that coping mechanisms and emotional responses are two strong components of psychotherapeutic approaches and are often tied together, considering that coping mechanisms involuntarily manifest when strong emotions are involved [46]. The overlap of these two clusters might therefore indicate that interactions reflecting coping mechanisms could be further detailed as per other characteristics. For example, coping mechanisms found in the green cluster might be more tied to *aspirations* and *beliefs about voices and schizophrenia*, whereas the ones found in the yellow cluster might be more tied *to emotional responses*.

### Limitations

While using the k-means algorithm enabled clustering, a larger dataset would have been preferred to account for the errors linked to the centroids of the clusters being dragged by interactions that are outliers. It should be noted that the transcripts examined in our research were typed in Canadian French, and locating vectorizers which included stopwords was not possible to for that language. As insignificant terms can be considered part of a word vector, the accuracy may have been impacted. Another limitation was the small sample of patients involved in the presented study as it affected the generalizability of the study considering that the interactions identified were part of a small number of participants.

## 5. Conclusions

Unsupervised machine learning can be a beneficial approach in the mental health field, bringing an objective evaluation of verbatims of AT. Our study allowed the identification of three major clusters of interactions for the avatar’s interactions and four major clusters for the patient’s interactions. As compared to the previously established qualitative analysis realized by human coders on the same dataset, it was observed that the results for the clustering of avatar interactions were similar to the ones identified by human coders. However, there was a greater divergence for the patient interactions, which were scattered across the identified clusters. The interactions previously labeled coping mechanisms and aspirations were the two types that were mainly classified together, whereas the other labels were more heterogeneously scatted across the four clusters. This study was the first attempt to conduct unsupervised machine learning on AT and provides quantitative insight into the inner interactions taking place during immersive sessions. The consideration of further data, such as the addition of emotions or psychomotor indices, could be beneficial to better comprehend the inner processes of AT and evaluate its implication in regard to the therapeutic outcome.

## Figures and Tables

**Table 1:** Main themes (labels) and number of text files for the labeled dataset.

Main Themes (Avatar)	Number of Text Files (n)	Percent of Text Files (%)
Confrontational techniques	427	46.31
Positive techniques	495	53.69
Main Themes (Patient)	Number of Text Files (n)	Percent of Text Files (%)
Self-perceptions	132	11.58
Aspirations	260	22.81
Emotional responses	192	16.84
Coping mechanisms	356	31.23
Beliefs about voices and schizophrenia	200	17.54

**Table 2:** Main themes (labels) and number of text files for the unlabeled dataset.

Cluster Colors (Avatar)	Mapping with the Labeled Data Set	Number of Text Files (n)	Percent of Text Files (%)
Blue	Confrontational techniques	167	18.11
	Positive techniques	72	7.81
Green	Confrontational techniques	211	22.89
	Positive techniques	127	13.77
Red	Confrontational techniques	49	5.31
	Positive techniques	296	32.10
Cluster Colors (Patient)	Mapping with the Labeled Data Set	Number of Text Files (n)	Percent of Text Files (%)
Blue	Self-perceptions	12	1.05
	Aspirations	8	0.70
	Emotional responses	6	0.53
	Coping mechanisms	2	0.18
	Beliefs about voices and schizophrenia	10	0.88
Yellow	Self-perceptions	83	7.28
	Aspirations	95	8.33
	Emotional responses	109	9.56
	Coping mechanisms	79	6.93
	Beliefs about voices and schizophrenia	43	3.77
Green	Self-perceptions	33	2.89
	Aspirations	119	10.44
	Emotional responses	67	5.88
	Coping mechanisms	237	20.79
	Beliefs about voices and schizophrenia	144	12.63
Red	Self-perceptions	4	0.35
	Aspirations	38	3.33
	Emotional responses	10	0.88
	Coping mechanisms	38	3.33
	Beliefs about voices and schizophrenia	3	0.26

## Data Availability

The data presented in this study are available on request from the corresponding author. The data are not publicly available due to patients’ privacy.
